# Seasonal habitat preference and foraging behaviour of post-moult Weddell seals in the western Ross Sea

**DOI:** 10.1098/rsos.220500

**Published:** 2023-01-25

**Authors:** Kimberly T. Goetz, Michael S. Dinniman, Luis A. Hückstädt, Patrick W. Robinson, Michelle R. Shero, Jennifer M. Burns, Eileen E. Hofmann, Sharon E. Stammerjohn, Elliott L. Hazen, David G. Ainley, Daniel P. Costa

**Affiliations:** ^1^ Marine Mammal Laboratory, Alaska Fisheries Science Center, National Marine Fisheries Service, National Oceanic and Atmospheric Administration, 7600 Sand Point Way NE, Seattle, WA 98115, USA; ^2^ Department of Ecology and Evolutionary Biology, University of California Santa Cruz, 100 Shaffer Road, Santa Cruz, CA 95060, USA; ^3^ Center for Coastal Physical Oceanography, Old Dominion University, 4111 Monarch Way, 3 floor, Norfolk, VA 23508 USA; ^4^ Center for Ecology and Conservation, University of Exeter, Penryn TR10 9FE, UK; ^5^ Biology Department, Woods Hole Oceanographic Institution, 266 Woods Hole Road, Woods Hole, MA 02543 USA; ^6^ Department of Biological Sciences, Texas Tech University, Box 43131, Lubbock, TX 79409, USA; ^7^ Institute of Arctic and Alpine Research, University of Colorado, Campus Box 450, Boulder, CO 80309-0450, USA; ^8^ Environmental Research Division, Southwest Fisheries Science Center, National Oceanographic and Atmospheric Administration, 99 Pacific Street, Suite 255A, Monterey, CA 93940, USA; ^9^ H.T. Harvey and Associates Ecological Consultants, 983 University Avenue, Building D, Los Gatos, CA 95032, USA

**Keywords:** Weddell seal, distribution, seasonal movement, habitat, foraging behaviour, Ross Sea

## Abstract

Weddell seals (*Leptonychotes weddellii*) are important predators in the Southern Ocean and are among the best-studied pinnipeds on Earth, yet much still needs to be learned about their year-round movements and foraging behaviour. Using biologgers, we tagged 62 post-moult Weddell seals in McMurdo Sound and vicinity between 2010 and 2012. Generalized additive mixed models were used to (i) explain and predict the probability of seal presence and foraging behaviour from eight environmental variables, and (ii) examine foraging behaviour in relation to dive metrics. Foraging probability was highest in winter and lowest in summer, and foraging occurred mostly in the water column or just above the bottom; across all seasons, seals preferentially exploited the shallow banks and deeper troughs of the Ross Sea, the latter providing a pathway for Circumpolar Deep Water to flow onto the shelf. In addition, the probability of Weddell seal occurrence and foraging increased with increasing bathymetric slope and where water depth was typically less than 600 m. Although the probability of occurrence was higher closer to the shelf break, foraging was higher in areas closer to shore and over banks. This study highlights the importance of overwinter foraging for recouping body mass lost during the previous summer.

## 1. Introduction

Predators respond to prey availability, which can change according to complex interactions between prey life history and environmental features, and this response can also be somewhat affected by physiological preferences of the predator for certain prey as well as migratory routes. Within this context, optimal foraging theory predicts that organisms maximize fitness by behaving in ways that increase foraging efficiency (energy gained versus energy expended) [1]. Therefore, predators should adjust their movements to correspond with areas of maximum prey availability. For example, foraging organisms are likely to spend more time in areas where prey are abundant and easily caught, as exhibited by noticeable changes in horizontal movement such as increased turn angles and decreased travel speeds. In the absence of actual measures of the preyscape, these behavioural changes are referred to as area-restricted searches (ARSs), a proxy for areas of aggregated prey; thus, resulting in increased search activity in a given area [2]. Quantifying the movement and preferred habitat characteristics of marine mammals is challenging because they occupy three-dimensional space (with time being a fourth dimension), in which they cannot be easily observed by humans.

Decades of at-sea observation and surveys have shown that upper trophic level marine predators often target areas where oceanographic features such as current boundaries, frontal systems, seamounts and continental shelf breaks alter the horizontal as well as vertical water column structure features (i.e. thermoclines, haloclines and pycnoclines) that are known to positively affect prey availability [3–6]). These oceanographic features tend to aggregate prey, thus facilitating predator foraging efficiency [7–9]. For many marine predators, persistent regions of predictably high prey availability are essential for reproduction and survival [10]. Where prey aggregations are ephemeral, dependent on time scale, predators must be able to associate prey with certain environmental cues.

The Southern Ocean is home to six species of pinnipeds: Antarctic fur seals (*Arctocephalus gazella*), crabeater seals (*Lobodon carcinophagus*), Weddell seals (*Leptonychotes weddellii*), Ross seals (*Ommatophoca rossii*), leopard seals (*Hydrurga leptonyx*) and southern elephant seals (*Mirounga leonina*) [11]. For the so-called ‘ice seals’ (crabeater, Weddell, Ross and leopard seals), sea ice provides a platform on which to rest, breed and pup. However, a changing climate is affecting sea-ice extent and persistence in the high-latitude Southern Ocean [12–15]. In the Bellingshausen and Scotia seas, earlier retreat and later sea-ice advance has resulted in a sea-ice season that is two-three months shorter (compared to 1979–1980). By contrast, the Ross Sea sector of the Southern Ocean overall has been exhibiting later ice-edge retreat in spring and earlier ice-edge advance in autumn, resulting in a longer sea-ice season, particularly in offshore areas [13,16,17]. Near the coast, however, there are latent heat polynyas, which expand and contract throughout the winter-spring periods in response to katabatic winds blowing off the adjacent continent and Ross Ice Shelf [13,16,18]. Surface waters associated with these polynyas often have higher biological production due to early exposure to sunlight in the spring [19], thus resulting in favourable foraging conditions for marine mammals and seabirds throughout the ice season [20,21]. Clearly, ice seals have been coping with a changing environment [22–24], and their foraging behaviour is likely to provide clues to the behavioural plasticity that allow them to succeed in the face of seasonal and longer term climatological shifts. The very well-studied Weddell seal can offer us insights into this adaptability.

### 1.1. Weddell seal life history

Weddell seals reside year-round in Antarctic waters and, with recorded dives greater than 600 m, they are the second deepest diving phocid after the southern elephant seal [25]. Basic information on marine aspects of their existence has been accumulated over the past few decades (e.g. [26–35]). Weddell seals are thought to feed primarily on Antarctic silverfish (*Pleuragramma antarcticum*), with other prey species including Antarctic toothfish (*Disssostichus mawsoni*), icefish (*Neopagetopsis ionah*), *Trematomus* species, cephalopods and invertebrates [36–39]. The fact that seals prey on toothfish, which is also a silverfish predator, results in a complex interaction termed ‘intraguild predation’ [40].

Weddell seals form colonies at established locations on the coastal fast ice (ice held in place by grounded icebergs, capes and islands) where they give birth and raise pups during October–November (austral spring); remaining on the fast ice, they then breed and moult during January–February (austral summer). The four months that Weddell seals spend on the fast ice are energetically costly, with females losing approximately 38% of their body mass during lactation alone [41,42] and males also losing body mass while defending access to females [43]. During this period, Weddell seals are depleting prey within foraging range of their haul-outs (summarized in [40] and further detailed in the discussion). Although Weddell seals are considered capital breeders, that is, relying largely on fat reserves accumulated prior to pupping/breeding, both males and females forage sporadically during the reproductive season [39,42,43]. In other words, it appears that Weddell seals adjust their behaviour to balance physiological demands with the local availability of prey resources. However, the period of overwinter foraging (February–September, when they are no longer aggregated at haul-out sites) is critical for Weddell seals to recoup body mass and condition.

Due to their accessibility and relatively docile nature, Weddell seal physiology and demography have been studied extensively since the 1960s [25,37,44–46], with research extending as far back as the early 1900s [47]. Within that effort, several Weddell seal populations have been studied around Antarctica (Atka: [48]; Weddell Sea: [38,49,50]; Dumont D'Urville: [51]; Prydz Bay: [52–54]). Recently, the Ross Sea, as well as the circum-Antarctic population, has been quantified [55,56] with the most intensely studied population of Weddell seals being located in McMurdo Sound. For the latter, research has focused on behaviour, physiology and ecology primarily during the austral spring and summer. Within that effort, foraging studies have focused on broad-scale movement and dive behaviour [30,57–59], including fine-scale, three-dimensional tracking using acoustics devices or accelerometers [60–64]. That effort has occurred contemporary with studies of population dynamics [33,44,45,65,66], physiology [31,41,67–71] and prey capture [72–74]. By contrast, little has been learned about Weddell seal habitat use or foraging behaviour when away from breeding-moulting periods, that is, during the eight months when they remain within the Ross Sea pack-ice. Previous to present work, Testa [34] showed that some Weddell seals from the western Ross Sea (WRS) travel north during the fall and into the winter. Since then, a study by Harcourt *et al*. [75] is the only one to have examined winter foraging behaviour and which found general behavioural similarities between Weddell seal populations at three locations (Prydz Bay, Terre Adélie and the Ross Sea) that the authors attributed to available habitat.

### 1.2. Ross Sea oceanography

The Ross Sea contains the broadest continental shelf in the Southern Ocean, consisting of numerous banks and troughs running north to south, three latent heat polynyas, two sensible heat polynyas and extensive ice shelves [76]. The Ross Sea is entirely ice-free during most austral summers, depending on whether the ice breaks out of the extreme southern portion of McMurdo Sound, and, except for polynyas, is 100% ice-covered during austral winter. The polynyas, especially the Ross Sea and McMurdo Sound polynyas, are areas of high sea-ice production beginning in early autumn (late February and March) and contribute to the northward advancement of the pack-ice, including along the Victoria Land coast and northward into the adjacent Southern Ocean [77,78]. Sea-ice extent continues to increase until July–September, depending on the year, extending out to the Southern Boundary of the Antarctic Circumpolar Current, and then retreating southward until mid-February [79]. The fast ice in McMurdo Sound remains in place into January, briefly disappearing during late-January into March [80].

The Ross Sea polynya, driven by katabatic winds, is bordered by the winter pack-ice to the north [78]. With the arrival of sunlight in spring, phytoplankton begin to grow rapidly in the nutrient-rich open waters of the polynya, the latter made possible by the transport of warm, nutrient-rich Circumpolar Deep Water (CDW) onto the Ross Sea continental shelf through the deep troughs. Once on the shelf, this water mass now known as modified CDW (or mCDW) creates relatively warm subsurface waters in both spring and winter [81]. Where mCDW upwells along the shelf break, sensible heat polynyas can also appear. The nutrients supplied by mCDW promote primary productivity in the Ross Sea which leads to the production of phytoplankton that supports many marine species, including silverfish, an important prey item for Weddell seals [82–84]. By December or January, the maximum phytoplankton growth rate is reached within the polynya and, at this point, the Ross Sea is the most productive region in the Southern Ocean [78,85,86].

### 1.3. Outline of this study

Clearly, a tremendous amount of information exists about Weddell seals and their habitat. However, much more can be learned by linking seal movement and foraging behaviour with their habitat. Our study examines seasonal habitat preferences and foraging behaviour of Weddell seals in the WRS using biologgers and remotely sensed data. Specifically, our goals were to model both Weddell seal seasonal habitat and foraging behaviour in relation to environmental variables as well as to model seasonal foraging behaviour in relation to various dive parameters. Using a three-year dataset spanning from the end of January to the end of November, our models provide the first year-round seasonal description of Weddell seal habitat preference and foraging behaviour. Additionally, this study provides insight into how an upper-level predator may respond behaviourally to changing population size as well to an altered ocean due to climate change [24,40,87]. The Weddell seal is known as an ‘indicator species’ as specified by the Commission for the Conservation of Antarctic Marine Living Resources [88,89], particularly in regard to managing the Ross Sea Region Marine Protected Area. Given that this study increases our understanding of Weddell seal habitat preference and foraging behaviour, this iconic seal is even more invaluable as an indicator species for better managing this critical Marine Protected Area in the Ross Sea.

## 2. Methods

### 2.1. Animal capture and handling

**Figure 1.**
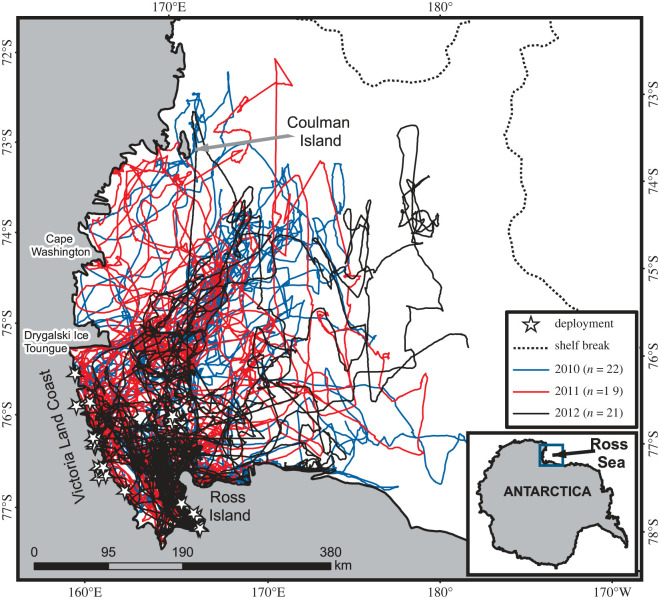
Weddell seal tracklines in the WRS during 2010 (blue), 2011 (red) and 2012 (black). Animals were tagged around Ross Island (*n* = 21) and along the Victoria Land coast (*n* = 41) with deployment locations denoted by stars. The dotted line represents the shelf break, or 1000 m bathymetric contour.

Between January and February 2010–2012, we deployed 62 Satellite Relay Data Loggers (SRDL), developed by the Sea Mammal Research Unit (SMRU Ltd, Scotland), on 11 male and 51 female Weddell seals. Fieldwork was conducted from McMurdo Station, Antarctica, with tag deployments occurring around Ross Island (*n* = 21) and along the adjacent Victoria Land coast north to the Drygalski Ice Tongue (*n* = 41) (figure 1).

Weddell seals were chemically immobilized with an intramuscular injection of a tiletamine HCL/zolazepam HCL mixture (0.5 mg kg^−1^). Twelve minutes post-injection, animals were captured using a hoop net. Subsequent intravenous injections containing a combination of ketamine hydrochloride and diazepam were administered intravenously, when necessary, to maintain immobilization. Tags were glued to the head of each seal with five-minute epoxy (Devcon® or Loctite® brand).

### 2.2. Environmental data

Parameters considered in this analysis include sea-ice concentration, distance to 10% ice concentration (i.e. boundary of a given polynya or access to open water pockets), bathymetric depth, bathymetric slope, distance to the continental shelf break, or 1000 m isobath, distance to the coast, mCDW at 150 m and mixed layer depth (MLD). The methods for how these parameters were obtained are described in the following paragraphs.

To assess the importance of ice concentration, we used daily Advanced Microwave Scanning Radiometer (AMSR-E or AMSR2) sea-ice concentration data with a 6.25 km resolution from the University of Bremen (http://www.iup.uni-bremen.de/seaice/amsr/, accessed January 2010–January 2014). Because daily sea-ice concentration values were stored in byte format (0 to 200), we used the raster calculator tool in ArcGIS 10.1 (Environmental Systems Research Institute) to convert ice concentration data to per cent ice cover. We also calculated distance to 10% ice concentration contours using the ‘Spatial Analyst’ extension in ArcGIS to assess proximity to open water.

Bathymetric depth was obtained from ETOPO-1, a one arc-minute global relief model of the Earth's surface [90] (http://www.ngdc.noaa.gov/mgg/global/, accessed April 2013). From bathymetric depth, we determined bathymetric slope using the ‘*Slope*’ tool in ArcGIS Pro which uses a three by three cell moving window to calculate the degree change in depth from one cell to the next. The 1000 m bathymetric contour was used to denote the continental shelf break [91] and the ‘Spatial Analyst’ extension in ArcGIS 10.1 was employed to create a distance surface representing distance to the shelf break. Note: depths of 1000 m also occur in the inner shelf, owing to isostatic depression of the continent due to its icecap, but these were ignored. In addition, a high-resolution coastline, Global Self-consistent Hierarchical High-resolution Shorelines [92], was used to create distance to the coastline.

A regional coupled circulation sea-ice numerical model for the Ross Sea by Dinniman *et al*. [93] provided daily estimates from 2010 through 2012 of water temperature, salinity and the location of mCDW. The model had 5 km horizontal resolution and included 24 vertical layers, with a thickness that varied with water column depth but was focused towards the top and bottom surfaces (e.g. for a typical Ross Sea continental shelf depth of 500 m, the maximum mid-water column layer thickness was 40.47 m, while the top and bottom layers were 4.97 m and 6.32 m thick, respectively) [93]. The model output (depth, pressure, salinity and temperature) was interpolated to a 1 m vertical resolution. We used the seawater toolbox (http://www.cmar.csiro.au/datacentre/ext_docs/seawater.htm*,* accessed March 2010) to calculate seawater density [94,95]. Density was used to calculate MLD, the depth where a 0.01 kg m^−3^ density difference from surface values was first detected [96]. Finally, we obtained the simulated distribution of mCDW at 150 m, the average dive depth for all tagged seals.

### 2.3. Tracking and diving data

Position estimates of tracking data obtained from ARGOS were filtered using a basic speed filter to remove unrealistic locations (i.e. locations resulting in a maximum horizontal speed greater than 15 km h^−1^ were removed). Weddell seal positions were interpolated at 2 h intervals using a forward-looking particle filtering model [97], which accounts for the errors associated with each ARGOS location class. Finally, tracklines were truncated based on the first and last transmitted dive times. One tag was eliminated from all dive analyses due to partial tag failure that resulted in animal location data without associated dive data.

Using the Marine Geospatial Modelling Environment (Version 07.2.1), we created 50 correlated random walks (CRWs) for each animal by making random draws from the distribution of angles and step lengths between subsequent points along each track [98]. Each CRW had the same number of steps as the corresponding seal track to facilitate comparisons. Similar to the methods in Hazen *et al*. [99], we randomly selected three of the 50 CRW to represent the possible behaviour of each animal, unbiased by environmental drivers. For this analysis, points along an animal's track were categorized as ‘present’ (where an animal was tracked) while those along each CRW were categorized as ‘absent’ (available habitat where an animal could have been based on movement parameters).

To determine probable foraging areas along each track, we identified ARSs by calculating first passage time (FPT), a measure of search effort. FPT is defined as the time required for an animal to cross a circle of a given radius [2] and was calculated for each track after removing all haul-out periods and data gaps longer than a week. We used a modification of the Fauchald & Tveraa [2] method in which the circle radius associated with the peak log variance was determined separately for each animal to account for individual variability [100]. After examining the average log variance in FPT for every 1 km increase in radii ranging from 1 to 50 km, we determined that Weddell seals on average operate on a 3 km scale. Using this scale, we calculated FPT for every location along the track for each animal.

Using R statistical software version 3.0.2 [101], the extract function within the ‘*raster*’ package (v. 3.4.13) [102] was used to sample values for each presence and absence location for the eight environmental covariates. Because ice concentration, distance from the 10% ice concentration contour and MLD data were available daily, these values were extracted for each unique date along the tracks. Locations with null values for any of the eight environmental variables were eliminated from the dataset.

Dive locations were determined by linking dive time with time along the trackline and linearly interpolated to the nearest minute. Due to ARGOS bandwidth limitations, tag transmissions of vertical dive profiles were limited to the four inflection points that provided the best fit for each dive. Once downloaded, dive data were filtered to eliminate erroneous data; dives were excluded from further analysis if: maximum dive depth was less than 5 m or greater than 1.5 times the known Ross Sea maximum bathymetry, dive duration was less than 10 s or greater than 5400 s, there were duplicate time stamps throughout the dive, vertical dive rate was greater than 4 m s^−1^, and time stamps decreased with increasing dive depth. Using the ‘Interp1’ function in MATLAB [103], each dive was interpolated at one-second intervals between inflection points. Finally, we calculated the following metrics for each dive: maximum dive depth (m), dive duration (min), bottom duration (time spent at depths greater than or equal to 80% of the max dive depth, min), descent rate (ms^−1^) (between the beginning of the dive to the first inflection point) and per cent depth within the water column (maximum dive depth relative to bathymetric depth with 0% = surface and 100% = seafloor).

Each track and dive location was categorized into one of the four seasons, delineated using the equinox and solstice dates (e.g. June 21–September 22 denotes winter). Note that the number of days and individuals in each season is a function of tag deployment duration (i.e. summer and spring consisted of fewer days since this analysis included data from deployments spanning from after the moult until returning to the colony before breeding and pupping commenced).

We calculated population means (average of individual means) and s.d. for the eight environmental variables and five dive parameters per season. In addition, we used the ‘*coin*’ package (version 1.4.2) [104] in R 3.0.2 to run Wilcoxon signed-rank tests, a non-parametric version of the *t*-test, to compare: (i) mean values of each environmental variable for seal presence and absence, (ii) paired mean values of each environmental variable for each season using the 25 individuals with data across all seasons and (iii) paired mean values of each dive variable per season using the 30 individuals with data spanning all seasons. Finally, the ‘*ggplot2*’ package in R was used to create violin plots (a combination of a boxplot and a kernel density plot) to further examine differences between Weddell seal presence and absence for each environmental variable.

To determine the appropriate spatial extent for model predictions, we used the kernelUD function within the ‘*adehabitatMA*’ package (v.0.3.14) [105] to produce seasonal utilization distribution (UD) kernels of the tracking data. A 6.5 km grid size and a 20 km smoothing parameter (or bandwidth) was used to produce each kernel. We then calculated the 50%, 75%, 95% and 100% UD. The 100% data contour encompassed all locations where an animal spent time.

### 2.4. Habitat suitability and foraging models

To assess collinearity between environmental variables, we used the ‘*corplot*’ package (v.0.84) [106] to calculate the Pearson's correlation coefficient [107,108]. For correlations greater than 0.70, only one of the two correlated variables was included in the model. Spatial autocorrelation was also examined for each seasonal dataset using the Moran.I function within the ‘*ape’* package [109] in R. This function computes the Moran's I autocorrelation index described by Gittleman & Kot [110] and showed no evidence of spatial autocorrection in any of the seasonal datasets used to model habitat suitability and foraging behaviour.

Due to their ability to handle non-parametric data and model nonlinear relationships typical of complex environments, generalized additive mixed models (GAMMs) were used to fit and predict the probability of Weddell seal presence and foraging behaviour. We used the ‘*mgcv*’ package (v.1.8.33) [111,112] to run each GAMM, which, unless otherwise specified, included a cubic regression spline, with shrinkage and five knots for all environmental variables. By shrinking the degrees of freedom to zero for each variable judged to be unimportant to the model, the shrinkage term provides an effective way of removing variables [113]. Animal ID was included as a random effect in all models to account for individual animal behaviour. Each initial GAMM was fit using all uncorrelated environmental variables for each season. Variables that were not significant at the *p* = 0.05 level were removed from the model using a backwards elimination approach until only significant variables remained in the final model for each season. To examine model fit, we produced diagnostic plots using the ‘*mgcViz’* package [114] in R (see electronic supplementary material, figure S1).

#### 2.4.1. Habitat suitability models

Data for habitat models were subset to include: (i) the unique cells per day (i.e. if an animal stayed in the same cell the entire day—it was included only once), and (ii) the absence points from CRWs that were not in the same cell as presences on the same day. Because the number of absence points from CRWs was greater than the number of presences, absence points were down-weighted so that the sum of their total was equal to the total number of presences (table 1). A training dataset consisting of 75% of the presence and absence data, chosen at random for each season, was used to fit a GAMM with a logistic link to determine the probability of Weddell seal occurrence relative to environmental variables [112,115]. In these models, the effects of the predictor variables are additive [116] and follow the form:

2.1 $$P_{i_{j}}=\frac{\exp[\beta_{0}+\Sigma_{i}f_{i}(x_{i})]}{1+\exp[\beta_{0}+\Sigma_{i}f_{i}(x_{i})]},$$

where *P_i_* is the probability of presence for each seal *j*, *β_0_* is the intercept to be estimated by the model and *x is* the value of the *i*th explanatory variable whose function *f_i_* is to be estimated.

**Table 1.** Mean and s.d. of eight environmental variables: ice concentration (%), distance to the 10% ice concentration contour (km), distance to the coast (km), distance to the continental shelf, or 1000 m isobath (km), bathymetric depth (m), bathymetric slope (degree), modified circumpolar deep water at 150 m (index) and MLD (m). Results are shown for both Weddell seal (presence) and CRW (absence) locations. Data were analysed separately for each season and mean values represent population means obtained by averaging individual means. Italicized indicates a significant difference between present and absent values at the *p* ≤ 0.05 level for a given environmental value, within a season, obtained from Wilcoxon signed-rank tests. Note that because tags were deployed late-January and typically ceased working by mid-November, data for summer and spring were slightly truncated.

	summer		fall		winter		spring	
	presence	absence	presence	absence	presence	absence	presence	absence
no. of (training)	3869	8639	11 407	29 107	5613	15 994	1744	4908
no. of individuals	49		58		48		30	
ice concentration	*49.8* *±* *17.3*	*41.1* *±* *17.1*	*87.9* *±* *11.6*	*80.7* *±* *9.1*	92.6 ± 9.7	92.7 ± 4.7	88.9 ± 12.6	87.1 ± 8.4
distance to 10% ice concentration	41.2 ± 28.8	38.3 ± 16.4	390.7 ± 282.9	401.1 ± 305.7	606.6 ± 307.6	626.5 ± 329.9	402.9 ± 290.9	420.5 ± 297.2
distance to coast	26.4 ± 25.1	18.7 ± 9.5	*51.4* *±* *42.2*	*24.0* *±* *11.4*	47.1 ± 39.3	28.8 ± 12.2	37.6 ± 24.6	26.7 ± 11.5
distance to shelf break	*491.1* *±* *85.1*	*539.8* *±* *26.0*	*441.7* *±* *91.3*	*528.2* *±* *34.1*	*443.1* *±* *98.3*	*520.8* *±* *36.9*	*478.4* *±* *65.2*	*521.9* *±* *35.2*
bathymetric depth	442.4 ± 143.1	479.6 ± 116.2	530.9 ± 95.8	516.8 ± 94.5	534.1 ± 113.3	528.5 ± 79.3	577.3 ± 136.6	534.3 ± 88.0
bathymetric slope	1.1 ± 0.7	0.9 ± 0.3	*0.7* *±* *0.4*	*0.8* *±* *0.1*	*0.7* *±* *0.7*	*0.7* *±* *0.1*	*0.6* *±* *0.4*	*0.8* *±* *0.2*
modified circumpolar deep water	17.3 ± 12.1	14.7 ± 12.0	23.5 ± 13.1	19.8 ± 12.5	23.8 ± 13.3	19.4 ± 11.4	21.2 ± 11.4	19.0 ± 11.0
MLD	34.3 ± 14.2	34.9 ± 14.3	115.5 ± 41.4	130.6 ± 48.9	*162.3* *±* *65.5*	*126.2* *±* *58.2*	111.6 ± 67.1	94.8 ± 52.4

For each of the four final models (one per season), we used a ‘receiver operating characteristic’ (ROC) curve to assess the diagnostic accuracy of the model [117,118]. ROC analysis measures how well a receiver is able to detect a signal in the presence of noise. In this case, a Weddell seal is either present or absent in a particular habitat unit and the ROC curve predicts a threshold at which the seal is present [119]. The optimal threshold represents the value at which errors of omission versus errors of commission are optimized. The ‘area under the curve’ (AUC) measures the discrimination ability of the model to correctly classify a Weddell seal as present or absent [120]. AUC values range from 0 to 1 with 0 indicating no discrimination, 0.5 no better than random chance and 1 indicating perfect discrimination ability [119]. Models with AUC values greater than or equal to 0.70 are considered ‘useful’ and those with AUC values greater than 0.9 are considered ‘very good’ because sensitivity is high relative to the false positive rate [121,122]. The ‘*pROC*’ (v.1.16.2) and ‘*cutpointr*’ packages (v.1.0.32) [123,124] were used to conduct the ROC and AUC analysis of the models. The performance of each GAMM was assessed using the AUC value created from the training dataset and an evaluation dataset which consisted of the remaining 25% of the presence/absence data not used in the training dataset.

Using the ‘predict’ function in the ‘*raster*’ package [102], we created predictions for each day spanning the duration of tracking data (29 January 2010–06 November 2012). Daily predicted probability of occurrence grids were averaged within each season to create a single probability surface. Each grid was then clipped to the extent of each respective season and rescaled from 0 to 1 to facilitate comparison. To convert daily predicted probability of occurrence to habitat suitability, we used the model-specific threshold value determined by maximizing the area under the ROC curve [125]. Raster cells above the threshold value were classified as 1, and all others as 0. Daily habitat suitability grids were then summed and 0 values were reclassified as NA. Finally, each seasonal habitat suitability grid was clipped to the extent of each respective season and rescaled from 0 to 1, thus reflecting the importance of each cell based on the number of days within each season classified as habitat.

#### 2.4.2. Horizontal foraging models

To examine foraging behaviour, we used the same location data as in the habitat models except haul-out periods and CRW data were removed. Data for foraging models were subset to include only unique cells per day, with the FPT value being summed across repeat cells (i.e. if an animal stayed in the same cell the entire day—it was included only once but the FPT value for each point within the repeated cells was summed). The response variable, FPT, was log-transformed and a GAMM for each season was fit to equation (2.2) using an identity link:

2.2 $$\log{\left(\mathrm{FPT}\right)}_{i_{j}}=\beta_{0}+\Sigma_{i}f_{i}(x_{i})\;\;(2)$$

where FPT*_i_* is the predicted FPT of an individual Weddell seal *j*, *β_0_* is the intercept to be estimated by the model and *x* is the value of the *i*th explanatory variable whose function *f_i_* is to be estimated.

Daily predictions of foraging were generated using the same methods as for habitat suitability. Daily predicted foraging grids were averaged within each season, clipped to the extent of each respective season and then rescaled from 0 to 1. To create an overall grid depicting the probability of foraging relative to probability of presence, we multiplied the predictive foraging surface and the probability of presence surface together for each season (i.e. highest values represent areas where animals are likely to be present and foraging). Finally, to depict areas where the most foraging was occurring when predicted seal presence was highest, we extracted cells with values greater than or equal to the median value of each seasonal grid. These areas were then mapped relative to the troughs and banks that characterize the underwater environment of the Ross Sea.

To understand how foraging behaviour changed throughout the year, we fit an additional GAMM using log FPT as the response variable and day of the year (DOY) as the explanatory variable. The GAMM included a cyclic cubic spine to allow for continuity at the endpoints, and the number of knots was unconstrained.

#### 2.4.3. Vertical foraging models

To understand the links between horizontal searching and dive behaviour, we used GAMMs to model the relationship between FPT and dive parameters (DDUR, BOTDUR, MXDEP, DRATE and PWC) for each season. Each dive was assigned an FPT value based on the encompassing 3 km FPT search radius. Only dives within this search radius were used in this analysis.

## 3. Results

Telemetry data showed that Weddell seals tagged near Ross Island and along the southern Victoria Land coast, in general, dispersed and travelled throughout the entire WRS but remained entirely in waters overlying the continental shelf (figure 1). There was a significant difference between Weddell seal presence and absence for distance to the shelf break and bathymetric slope for most seasons (table 1). Ice concentration in summer and fall also significantly differed between Weddell seal presence and absence locations, with Weddell seals occurring in areas with higher ice concentrations (table 1). Finally, in areas where Weddell seals were present, mean distance to the coast was significantly higher in fall and MLD significantly deeper in winter than locations where seals were not recorded (table 1).

**Figure 2.**
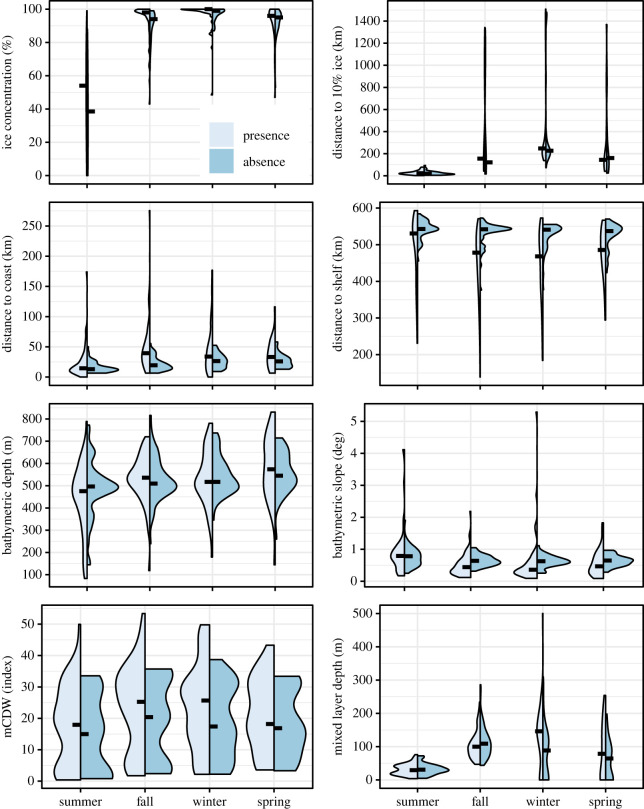
Violin plots showing the median and distribution of eight environmental variables by season: ice concentration (%), distance to10% ice concentration (km), bathymetric depth (m), bathymetric slope (degree), distance to the continental shelf, or 1000 m isobath (km), modified circumpolar deep water at 150 m (mCDW, index) and MLD (m). Each panel includes data for both Weddell seal (presence, light blue) and CRW (absence, dark blue) locations. Black horizontal lines show median values for each variable and the distribution of the data is indicated by the shape, representing kernel density plots.

When comparing mean values where Weddell seals were present for every two-way combination of seasons, nearly all environmental variables during summer were significantly different from those of the other three seasons—fall, winter and spring (table 2). Ice concentration, distance to 10% ice concentration, bathymetric depth and MLD were all significantly greater while bathymetric slope was significantly lower during fall, winter and spring than during summer (table 2). Finally, Weddell seals were significantly closer to the shelf break during fall than during summer and in waters with significantly shallower MLDs during spring than during winter (table 2). Median values show that Weddell seals were closer to the shelf break from fall to spring than during summer (figure 2). Across all seasons, median values for mCDW and distance to the coast were higher where Weddell seals occurred than to locations where seals could have been but were not observed (figure 2).

**Table 2.** Wilcoxon signed-rank test results comparing seasonal values between paired individuals for each environmental variable: ice concentration (%), distance to 10% ice concentration (km), distance to the coast (km), distance to the continental shelf break, or 1000 m isobath (km), bathymetric depth (m), bathymetric slope (degree), modified circumpolar deep water at 150 m (index) and MLD (m). Only Weddell seals with associated environmental data for all four seasons were included in this statistical analysis. Italicized and bolded *p*-values are significant at the *p* = 0.05 level, with italicized indicating a decrease between the two indicated seasons and bold indicating an increase in values between the two seasons.

	summer-fall		summer-winter		summer-spring		fall-winter		fall-spring		winter-spring	
	*v*	*p*	*v*	*p*	*v*	*p*	*v*	*p*	*v*	*p*	*v*	*p*
ice concentration	1	**<0.001**	0	**<0.001**	8	**<0.001**	91	0.055	148	0.711	237	*0.045*
distance to 10% ice concentration	0	**<0.001**	0	**<0.001**	0	**<0.001**	34	**<0.001**	120	0.264	183	0.597
distance to coast	41	**<0.001**	59	**0.004**	62	**0.006**	222	0.114	174	0.712	134	0.458
distance to shelf break	245	*0.026*	223	0.107	228	0.080	113	0.191	145	0.653	163	1.000
bathymetric depth	52	**0.002**	70	**0.012**	59	**0.004**	144	0.634	98	0.085	122	0.529
bathymetric slope	260	*0.007*	263	*0.006*	293	*<0.001*	198	0.353	211	0.200	191	0.458
modified circumpolar deep water	30	**<0.001**	94	0.067	74	**0.016**	207	0.241	176	0.731	100	0.096
MLD	0	**<0.001**	0	**<0.001**	25	**<0.001**	139	0.055	285	0.289	362	*0.007*

While bottom and dive duration were similar in summer and fall, durations increased in winter and spring (table 3). A similar trend was seen in maximum dive depth, in which Weddell seals dived deeper as the seasons progressed from summer through the following spring. In fact, maximum dive depth in spring was nearly twice that of summer despite diving to similar locations within the water column (PWC). In other words, seals occupied deeper waters in spring than in summer but were consistent in their preference of per cent water column depth. Comparing the same individuals across seasons, the majority of the five dive parameters were significantly different (table 4). Interestingly, values for all dive parameters in summer were significantly different from those of winter; dive duration, bottom duration and maximum depth increased while descent rate and the per cent water column decreased.

**Table 3.** Number of dives, number of individuals and mean ± s.d. of five Weddell seal dive metrics: dive duration (min), bottom duration (min), descent rate (ms^−1^), maximum dive depth (m) and per cent water column depth (%). Data were analysed separately for each season and mean values represent population means obtained by averaging individual means. Note that because tags were deployed late-January and typically ceased working by mid-November, data for summer and spring were slightly truncated.

	summer	fall	winter	spring
no. of dives	91 261	101 009	21 947	3013
no. of individuals	59	57	48	30
dive duration	8.6 ± 2.2	8.2 ± 2.0	10.0 ± 2.7	12.3 ± 3.9
bottom duration	3.8 ± 1.1	3.7 ± 1.2	4.4 ± 1.5	5.9 ± 2.1
descent rate	1.2 ± 0.2	1.2 ± 0.2	1.1 ± 0.2	1.2 ± 0.2
maximum dive depth	114.5 ± 36.7	128.0 ± 37.6	140.1 ± 51.7	223.5 ± 99.4
per cent water column depth	39.3 ± 11.6	28.4 ± 10.9	28.7 ± 11.8	39.3 ± 19.9

**Table 4.** Wilcoxon signed-rank test results comparing seasonal values between paired individuals for five dive metrics: dive duration (min), bottom duration (min), descent rate (ms^−1^), maximum dive depth (m) and per cent water column depth (%). Only Weddell seals with associated dive data for all four seasons were included in this statistical analysis (*n* = 30). Italicized and bolded *p*-values are significant at the *p* = 0.05 level, with italicized indicating a significant decrease between the two indicated seasons and bold indicating an increase in values between the two seasons.

	summer-fall		summer-winter		summer-spring		fall-winter		fall-spring		winter-spring	
	*v*	*p*	*v*	*p*	*v*	*p*	*v*	*p*	*v*	*p*	*v*	*p*
dive duration	271	0.440	46	**<0.001**	67	**<0.001**	26	**<0.001**	49	**<0.001**	112	**0.012**
bottom duration	241	0.871	68	**<0.001**	69	**<0.001**	57	**<0.001**	57	**<0.001**	106	**0.008**
descent rate	123	**0.023**	355	*0.011*	159	0.135	441	*<0.001*	236	0.952	118	**0.175**
maximum dive depth	94	**0.003**	44	**<0.001**	26	**<0.001**	99	**0.005**	44	**<0.001**	69	**<0.001**
per cent water column depth	−3.77	*<0.001*	3.55	*<0.001*	−0.22	0.839	0.03	0.984	−2.87	**0.003**	2.48	0.012

### 3.1. Habitat models

Distance to the shelf break and distance to the coast were highly correlated across seasons (−0.65 to −0.79). Therefore, only distance to the shelf was retained in seasonal habitat and foraging models. During fall and winter, all environmental variables were significant and explained 27% and 17% of the deviation in Weddell seal occurrence, respectively (table 5). During summer and spring, a single non-significant variable was dropped from each of the two models (MLD in summer and distance to 10% ice concentration in spring). The final model for summer and spring explained 25% and 14% of the deviation in Weddell seal occurrence, respectively. The AUC value for each of the final seasonal models ranged between 0.74 and 0.81, indicating that models performed better than chance and could distinguish presences from absences in at least 74% of the cases after accounting for model variables (table 5). AUC values generated from the training and evaluation datasets were similar, indicating limited overfitting to the data and increased transferability of the models to novel datasets.

**Table 5.** Model covariates, the number of presence (P) and CRW absence (A) points (training data), number of individuals (no. of Ind), deviance explained (Dev Exp—training data), *R*^2^ adjusted (training data), AUC values (training and evaluation data) and threshold value used in the final habitat models (H) to predict probability of occurrence and habitat from environmental parameters by season. Abbreviations for model variables are as follows: ice concentration (ICECON, %), distance to 10% ice concentration (DICE10, km), distance to the coast (DCOAST, km), distance to the continental shelf, or 1000 m isobath (DSHELF, km), bathymetric depth (BATH, m), bathymetric slope (SLOPE, degree), modified circumpolar deep water at 150 m (mCDW, index) and MLD (m).

model	significant variables	P/A	no. of Ind	Dev Exp	*R*2 Adj	AUC (train/eval)	threshold
H_SUMMER	ICECON, DICE10, DSHELF, BATH, SLOPE, mCDW	2884/6462	48	0.25	0.29	0.81/0.81	0.49
H_FALL	ICECON, DICE10, DSHELF, BATH, SLOPE, mCDW, MLD	8535/21 866	58	0.27	0.31	0.81/0.81	0.57
H_WINTER	ICECON, DICE10, DSHELF, BATH, SLOPE, mCDW, MLD	4193/11 973	48	0.17	0.20	0.76/0.76	0.52
H_SPRING	ICECON, DSHELF, BATH, SLOPE, mCDW, MLD	1298/3671	30	0.14	0.16	0.74/0.75	0.47

**Figure 3.**
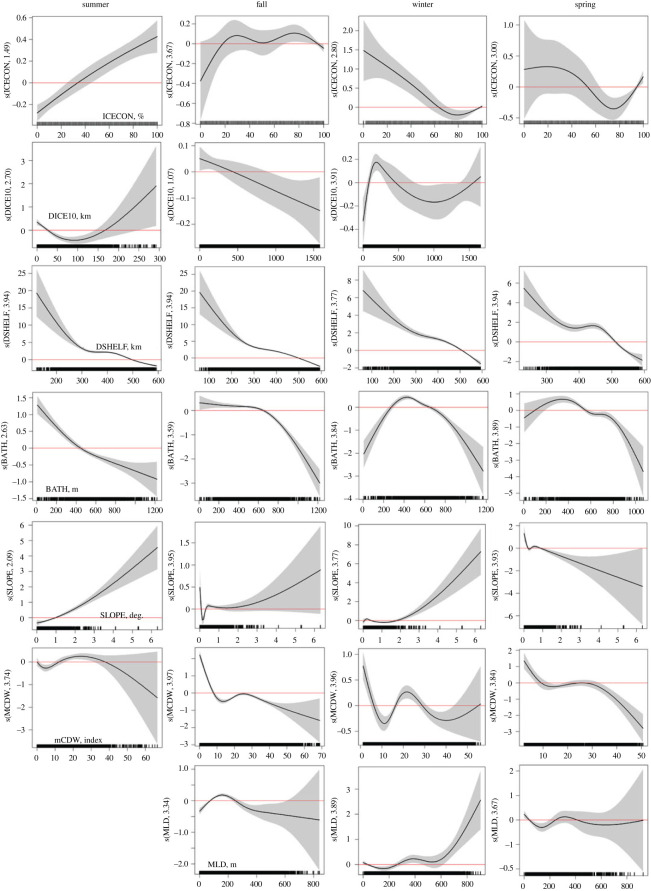
Probability of Weddell seal presence in the WRS predicted from a GAMM for each season. Plots show the relationship between Weddell seal occurrence and seven environmental variables: ice concentration (ICECON), distance to 10% ice concentration (DICE10), bathymetric depth (BATH), bathymetric slope (SLOPE), distance to the continental shelf break, or 1000 m isobath (DSHELF), mCDW and MLD. Shaded areas represent the 95% confidence interval. The effect of the explanatory variable on the response is on the logit scale where zero (solid red line) or negative numbers show no effect. Units for the *x*-axis are indicated on the first panel in each row. Missing plots indicate that the variable was not significant.

During summer, the probability of Weddell seal presence increased with increasing ice concentration (figure 3). However, the opposite pattern was found during winter and spring. From summer through fall, when not associated with breeding or pupping, Weddell seals preferred to be closer to the shelf break (figure 3). In spring, seals preferred higher bathymetric slope than in other seasons (figure 3). In summer Weddell seals preferred to be within 20 km or beyond 170 km of 10% ice concentration contour while in fall they preferred to be within 500 km. During winter, seals preferred to be either within 550 km or beyond 1,500 km from access to open water. Weddell seals preferred shallower waters during the summer (less than 400 m) and fall (less than 600 m) but this preference changed to waters ranging from approximately 200 to 600 m in winter and spring (figure 3). Generally, the probability of Weddell seal presence was highest when the mCDW index was less than 30 and during summer and winter, for values between approximately 15 and 30 (figure 3). In fall, winter and spring, Weddell seals preferred intermediate MLD values; approximately 100–250 m in fall, greater than 250 m in winter and approximately 250–400 m in spring (figure 3).

**Figure 4.**
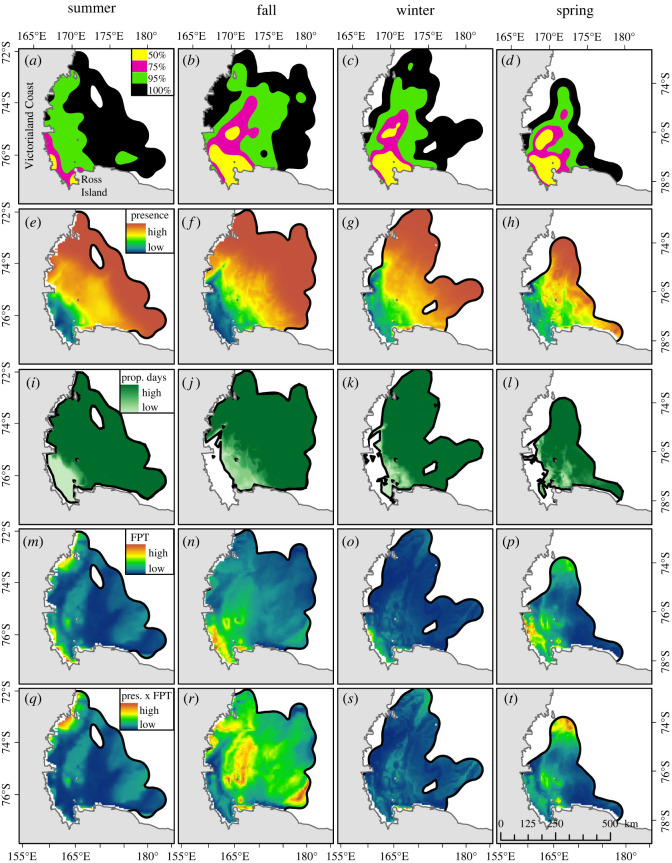
(*a–d*) The 50% (yellow), 75% (magenta), 95% (green) and 100% (black) UDs of Weddell seals in the WRS each season. (*e–h*) Seasonal probability of Weddell seal presence with warmer colours indicating higher probability of seal occurrence. (*i–l*) Preferred habitat colour scaled by the proportion of days per season that each cell was classified as ‘habitat’. (*m–p*) seasonally predicted Weddell seal foraging, as measured by FPT, and (*q–t*) predicted Weddell seal foraging in relation to the predicted probability of presence.

The north/south extent of the entire Weddell seal range, or 100% UD, was similar in summer, fall and winter and slightly reduced in spring (figure 4*a–d*). However, in fall, winter and spring, the 50%, 75% and 95% UDs were located more centrally and farther offshore than in summer (figure 4*a–d*). Across seasons, the predicted probability of Weddell seal occurrence was highest at the farthest extent of the range and lowest in McMurdo Sound and south of the Dyrgalski Ice Tongue along the Victoria Land coast (figure 4*e–h*). While it may appear counterintuitive that high-use areas (i.e. 50% UD) occur where predicted probability of Weddell seal presence is low, one must consider the fundamental differences between UDs and habitat preference. UDs are used to examine the relative frequency of occurrence and do not take into account environmental features while habitat preference is determined by statistically comparing habitat use and availability and is contingent on both samples.

The ROC threshold value for each habitat preference model was 0.49, 0.57, 0.52 and 0.47 for summer, fall, winter and spring, respectively (table 5). Preferred habitat across all seasons was located in the central WRS towards the continental shelf break (figure 4*i–l*). From fall through spring, non-preferred habitat was located in McMurdo Sound and north along the Victoria Land coast to Drygalski Ice Tongue (figure 4*i–l*). During the summer, these same areas were predicted as habitat for only one day (light green areas in figure 4*i*).

### 3.2. Horizontal foraging models

**Figure 5.**
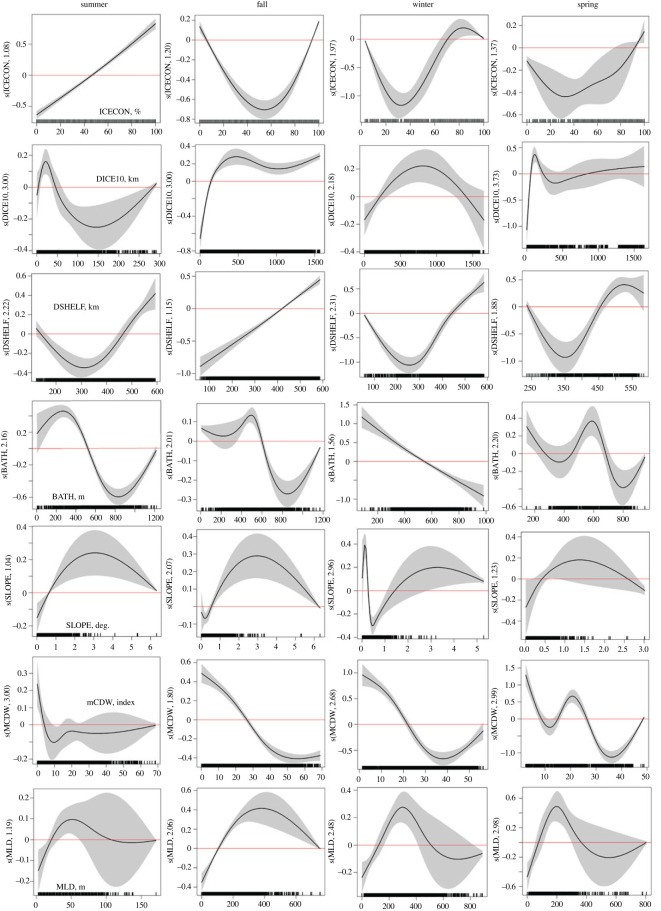
Probability of Weddell seal foraging in the WRS predicted by a GAMM for each season. Plots show the relationship between FPT and seven environmental variables: ice concentration (ICECON), distance to 10% ice concentration (DICE10), bathymetric depth (BATH), bathymetric slope (SLOPE), distance to the continental shelf break, or 1000 m isobath (DSHELF), mCDW and MLD. Shaded areas represent the 95% confidence interval. The effect of the explanatory variable on the response is on the log scale where zero (solid red line) or negative numbers show no effect. Units for the *x*-axis are indicated on the first panel in each row.

At the 3 km scale, all environmental variables were significant predictors of FPT during each of the four seasons (table 6). An increase in FPT was associated with increasing ice concentration above 40% in summer, indicating increased foraging behaviour (figure 5). However, FPT was highest for both low and high ice concentrations in fall, while in winter and spring, foraging was associated with high ice concentrations (80% and higher). Weddell seal foraging probability was highest closer to the 10% ice concentration contour in summer and spring but, in fall and winter, the probability of foraging was highest when seals were greater than 500 km from the 10% ice concentration contour (figure 5). Across all seasons, FPT was highest in water depths less than 600 m and when the mCDW index was less than 25. In the summer, FPT increased with MLD greater than 25 m in summer, between 100 and 300 m in fall and winter, and 100 and 200 m in spring (figure 5). Across seasons, foraging was highest farther from the shelf break, along the coast, and in areas of moderate bathymetric slope (figure 5).

**Table 6.** Model covariates, the number of data points (*n*), number of individuals (no. of Ind), deviance explained (training data), *R*^2^ adjusted used in the final horizontal (HORZ) and vertical (VERT) foraging (F) models to predict foraging behaviour (FPT) from environmental and dive parameters by season. Abbreviations for model variables are as follows: ice concentration (ICECON, %), distance to10% ice concentration (DICE10, km), distance to the coast (DCOAST, km), distance to the continental shelf break, or 1000 m isobath (DSHELF, km), bathymetric depth (BATH, m), bathymetric slope (SLOPE, degree), modified circumpolar deep water at 150 m (mCDW, index) and MLD (m).

model	significant variables	*n*	no. of Ind	Dev Exp	*R*^2^ Adj
F_HORZ_SUMMER	ICECON, DICE10, DSHELF, BATH, SLOPE, MLD, mCDW	3169	52	0.28	0.28
F_HORZ_FALL	ICECON, DICE10, DSHELF, BATH, SLOPE, MLD, mCDW	10 531	58	0.11	0.11
F_HORZ_WINTER	ICECON, DICE10, DSHELF, BATH, SLOPE, MLD, mCDW	4457	46	0.18	0.17
F_HORZ_SPRING	ICECON, DICE10, DSHELF, BATH, SLOPE, MLD, mCDW	1361	27	0.25	0.24
F_VERT_SUMMER	DDUR, DRATE, PWC	67 405	52	0.22	0.21
F_VERT_FALL	DDUR, DRATE, PWC	75 782	50	0.19	0.16
F_VERT_WINTER	DDUR, DRATE, PWC	17 928	42	0.05	0.05
F_VERT_SPRING	DRATE, PWC	2390	22	0.11	0.11

**Figure 6.**
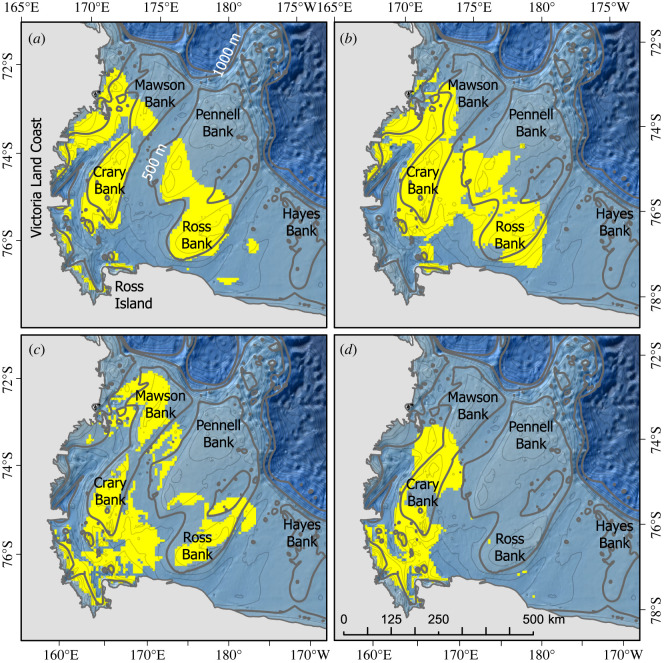
Predicted high-use foraging areas (greater than or equal to median FPT) where Weddell seals are present in relation to Ross Sea Banks in summer (*a*), fall (*b*), winter (*c*) and spring (*d*).

In all four seasons, foraging models predicted the highest FPT in the coastal vicinity of Ross Island and dispersed throughout the central WRS, particularly over Crary Bank which will be discussed in more detail later (figure 4*m–p*). In addition, models predicted high FPT values from Ross Island (77°S), north along the Victoria Land coast up to the Drygalski Ice Tongue (75°S, figure 4*m–p*). Seasonal predictions of Weddell seal occurrence (figure 4*e–h*) were combined with seasonal predictions of FPT (figure 4*m–p*) to identify areas most likely to be selected as foraging habitat (figure 4*q–t*). The highest predicted FPT within preferred Weddell seal habitat occurred around Ross Island (though difficult to see in the figure) and extended from the central WRS to the farthest extent of Weddell seal travel in each season (figure 4*q–t*). Note that the colour coding for winter foraging is biased towards the very few cells with high FPT predictions near Ross Island. For comparison, figure 6 shows areas where predicted values are greater than or equal to the median predicted FPT for each season. High FPT values are predicted over Crary Bank across all seasons, and over Mawson, Ross and Pennell banks during summer, fall and winter (figure 6).

**Figure 7.**
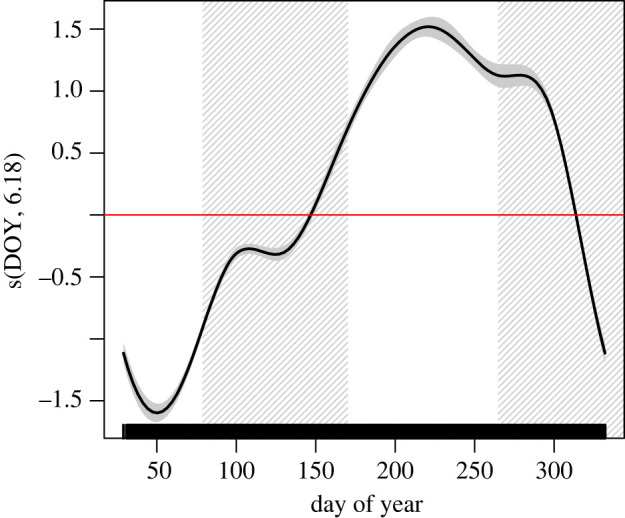
Smoothed function for DOY in predicting FPT, shaded and non-shaded areas represent seasons, from summer to spring, respectively. Shading along the fitted line represents the 95% confidence interval. The effect of the explanatory variable on the response is on the log scale where zero (solid red line) or negative numbers show no effect.

FPT varied markedly throughout the year with the lowest values being found in summer followed by a slight peak in fall, a continued increase until reaching an overall peak in winter and a gradual decline in spring (figure 7). DOY explained 35% of the variation in FPT.

### 3.3. Vertical foraging models

**Figure 8.**
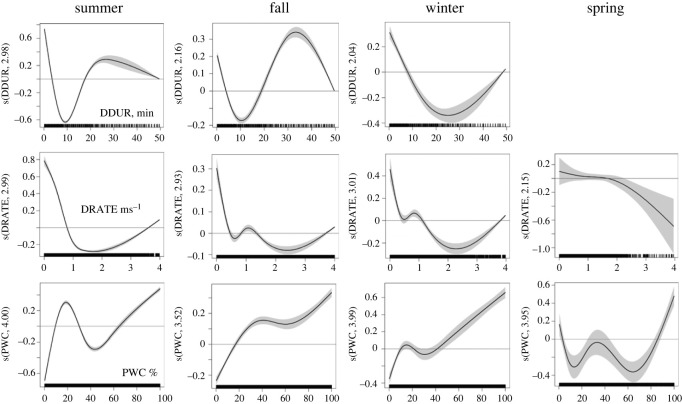
Results from the final GAMM were used to predict FPT for each season. Plots show the relationship between FPT for Weddell seals in the WRS and three dive metrics: dive duration (DDUR), per cent water column depth (PWC) and descent rate (DRATE). The shaded areas represent the 95% confidence interval. The effect of the explanatory variable on the response is on the log scale where zero (solid red line) or negative numbers show no effect. Units for the *x*-axis indicated on the first panel in each row. Missing plots indicate that the variable was non-significant.

Because maximum dive depth and per cent water column depth as well as dive and bottom durations were highly correlated (*r* > 0.70), both maximum dive depth and bottom duration were excluded from the models. GAMM results showed that dive duration, descent rate and per cent water column depth were significant in predicting FPT during summer, fall and winter, explaining 5–22% of the deviation in FPT (table 6). During summer and fall, FPT was highest when dive duration was less than 6 min or between approximately 20–30 min and during slow descent rates (less than 0.8 ms^−1^) (figure 8). During winter, FPT was greatest when dive duration was less than 6 min and when descent rates were less than 0.5 ms^−1^ (figure 7). Weddell seals generally exhibited increased FPT with increasing PWC from summer to winter, with peaks in FPT observed around 15% and greater than 60%. However, during spring, FPT was highest when seals were conducting dives either near the surface or near the bottom and when descent rates were low.

## 4. Discussion

Upon departure from the colony during late summer, a small proportion of Weddell seals tagged in our study remained near Ross Island year-round while the majority of animals spread widely to more northern habitat located within the remaining sea ice. Both Testa *et al*. [126] and Hindell *et al*. [60] experimentally showed that, owing to a limited foraging range and central-place foraging, Weddell seals deplete the prey around colonies during the breeding season [40,126,127]. The dispersal of seals hundreds of kilometres to the north and east of Ross Island would likely reduce intraspecific competition. The same phenomenon is exhibited around penguin colonies [128,129]. In late summer, Weddell seals preferred to occupy and forage in areas with higher slope, a pattern that indicates that animals are foraging coastally but near deep water, particularly between Cape Washington and Coulman Island as well as along the edges of banks. During this time, seals also preferred areas farther from land since the land-fast ice is no longer sturdy enough to reliably support them [130,131]. Overall, we found that the density of Weddell seals was highest adjacent to steep undersea topography in summer, which agreed with the findings of other studies [60,131]. Hindell *et al*. [60] hypothesized that fish were more concentrated in these areas due to enhanced upwelling associated with the topography. In that regard, LaRue *et al*. [131] hypothesized that with greater depth, there is greater water volume nearby, providing more prey and a wider array of foraging habitat. While these studies only considered short foraging trips during the breeding season, our study found similar results over a broader temporal scale—summer through the following spring.

During summer and winter, seals also foraged within the top 20% of the water column (pelagic). This agrees with several previous studies indicating that Weddell seals exploit both pelagic and bentho-pelagic prey such as Antarctic silverfish and toothfish [36,37,50,60,63,132]. Increased foraging was associated with slower descent rates and both shorter and longer dive durations. A more recent study showed that Weddell seals in the WRS spent less time near the bottom than those in Prydz Bay and Terre Adélie, but this was likely due to intrinsic factors such as age, sex and body-size [75].

During fall, seals continued to forage extensively near the coast, north of the Drygalski Ice Tongue, between Cape Washington and Coulman Island, but they also foraged heavily over the banks, farther from the coast—a pattern that is consistent with foraging in areas with heterogeneous habitat. As ice formation and sea cover progressed through fall and winter, Weddell seals preferentially occupied areas with open water access. However, foraging behaviour was higher in dense pack-ice, farther away from open-water pockets. These findings suggest that the under-ice and ice-edge environments play an important role in foraging, with prey resources located farther from areas that provide open-water access. Foraging within the dense pack-ice also provides refuge from predatory killer whales [127] that are known to frequent the Ross Sea [133].

During winter, Weddell seals continued to forage both coastally and over banks. However, unlike during fall, the coastal area between Cape Washington and Coulman Island was no longer frequented by foraging Weddell seals, a pattern possibly related to both intra- and interspecific competition. At the same time, Weddell seals are arriving in the vicinity to pup/breed [55,131]; emperor penguins are also returning to the largest colony occurring along the same section of coast and, therefore, also depleting prey resources in the area [134,135]. This is consistent with the findings of LaRue *et al*. [55] who showed that, while both species sought fast ice, the number of Weddell seals was inversely proportional to the number of emperor penguins in an area and vice versa. Instead, Weddell seal foraging areas in winter were located south of the Drygalski Ice Tongue, farther south than during previous seasons, where no penguin colonies are located [129]. While consistent with Harcourt *et al*. [75], our results showed that Weddell seals in the WRS had a much larger UD during winter, possibly due to a larger sample size or changes in environmental conditions between 2010–2012 and 2014–2019.

Sea ice in the WRS is at its greatest extent [79] in late winter, and its magnitude is positively correlated with higher Weddell seal recruitment to breeding colonies the following spring [136]. Weddell seals preferentially occupied areas with low to intermediate ice concentrations and foraging was highest where ice concentration was at or near 100%. Increased foraging in the dense pack-ice could be driven by the abundance of ice algae that use the under-ice environment, thus attracting cryopelagic predators such as *Pagothenia borchgrevinki*, a known prey item of Weddell seals in the WRS [73,137]. Weddell seal foraging in the dense pack-ice could also be driven by the presence of Antarctic toothfish, another important prey item, found above the bottom in dense ice cover [138]. Furthermore, krill abundance, especially crystal krill (*Euphausia crystallorophias*), is also positively correlated with ice cover [139], and krill is the primary prey item of Antarctic silverfish, an essential dietary item of Weddell seals [36,73,74,140].

During spring, before returning to breeding colonies, Weddell seals continued to forage coastally and preferred areas on top of or peripheral to banks or land for both habitat and foraging grounds. Unlike other seasons, distance to open-water pockets did not predict preferred habitat. La Rue *et al*. [131] found a ‘Goldie locks’ relationship in which the fast ice needed to be wide enough perhaps to protect from killer whales but not too wide, which may present a challenge with breath-holding capacity in reaching open water. However, seals preferentially foraged both near and far from open-water pockets. Weddell seal preference to forage near open water is likely related to the rapid opening of latent heat polynyas in the spring [79]. During the four-month period when Weddell seals are on the fast-ice pupping, breeding and moulting, they forage to a limited degree with potential prey items being associated with the sea ice (e.g. [127]). When seals leave the breeding colonies in summer, the marginal ice zone around the Ross Sea Polynya is highly productive, and this productivity has likely transferred up enough trophic levels to support a large predator, such as the Weddell seal [21].

Across all seasons, models showed that adult Weddell seals preferentially exploited the banks in the WRS and foraging behaviour was highest when seals were diving near or at the bottom (bentho-pelagic). Weddell seals in the WRS foraged extensively over Crary Bank across all seasons while Ross, Pennell and Mawson banks appeared to provide important foraging habitats from summer through winter. Seals foraged less extensively in troughs where the mCDW index was highest. However, the predominance of foraging in shallower waters with low to moderate mCDW index values indicates that Weddell seals may be targeting prey species on, or proximate to, banks while using ice-covered areas over troughs for resting or transiting.

The locations where mCDW intrudes onto the shelf are determined primarily by bathymetry [83]. This coupled with subsequent spreading of mCDW on the shelf and the melting of sea-ice influences patterns in the spring phytoplankton bloom in the Ross Sea [81,141,142]. The production and transport of organic material on the shelf affects prey available to benthic consumers [143]. Benthic communities are richest on the shoulders of banks, where currents bringing food particles to filter feeders are strongest [143]. Benthic productivity plays a large role in structuring both the water column and benthic portions of the ecosystem, with benthic organisms, principally invertebrates (and the fish that feed on them), depending on the influx of sinking organic debris [76,143]. Barry *et al*. [143] found that the mean percentage cover of animals (summed over all megafauna taxa) and megafauna density on the banks and crests to be six and four times greater than in the deeper areas between basins and troughs [143].

Foraging over the Ross Sea banks combined with diving in proximity to the bottom suggests that prey resources are more available over the banks. Crary Bank not only provides important foraging habitat for Weddell seals across seasons, but also offers foraging habitat for emperor penguins from the large Cape Washington colony, another silverfish predator [144]. Studies indicate that silverfish is a primary prey item of Weddell seals during summer [36,74]. However, it is not known whether summer diet indicates year-round foraging habits. Given the highly dynamic nature of the Ross Sea ecosystem throughout the year, it is likely that Weddell seals adapt to changes in the local abundance of prey species by altering their diet. When seal numbers in McMurdo Sound were restricted during a period of multi-year fast ice (free-board of ice cracks too high for seals to haul-out [24]), without seal predation, the benthic fish fauna changed from its usual composition [145]. Additional evidence of seal dietary adaptability is apparent in the Weddell Sea, indicating that while Antarctic silverfish is also the primary summer diet for those animals [38], in spring, silverfish was no longer present in their diet; instead prey items consisted of many other notothenioid fish including *Trematomus* species [38,50].

## 5. Conclusion

This study presents the first quantitative analysis of post-moult Weddell seal habitat preference and foraging behaviour across all four seasons in the WRS. We successfully modelled and predicted habitat preference and foraging behaviour using environmental variables, and modelled vertical foraging behaviour using dive parameters. Weddell seal foraging intensity in the summer is relatively low compared to the rest of the year, a pattern perhaps attributed to reproduction and moulting, and recovery therefrom, or possibly reduction of prey near haul-out aggregations (Storer-Ashmole's Halo) [146]. Weddell seals are considered capital breeders and rely primarily on stored body reserves during this time, with females losing nearly 40% of their body mass during lactation alone [41]. In defending access to females, males also do not forage and lose mass. Once pups are weaned, Weddell seals are no longer tied to the breeding colonies and can travel farther in order to recoup lost mass, making the fall, winter and spring the most important seasons for Weddell seal foraging. These results are supported by Shero *et al*. [71], who found that the overwinter foraging period was necessary for female Weddell seals to regain mass and body condition, despite limited foraging during the breeding and moulting periods.

The Ross Sea is the most productive region in the Southern Ocean and reaches farther south than any other marine system on Earth [147]. Nevertheless, significant annual variation in polynya size, sea-ice extent and productivity is exhibited [78]. Due to its vast shelf, complex submarine topography and productive polynyas, the Ross Sea is considered a biodiversity hotspot [148–150]. Included in that biota is the Weddell seal, more abundant in the Ross than anywhere else [55]. Herein, models showed the importance of the diverse habitats found in the WRS in predicting seasonal Weddell seal habitat and foraging behaviour.

Knowing how Weddell seals respond to predictable seasonal changes in their environment can provide insights into how their habitat or foraging behaviour will change with forecasted natural and anthropogenic climate variation. Although the Ross Sea ecosystem remains relatively intact, changes in hydrographic properties, such as salinity, and sea-ice extent have been documented in response to changing environmental and atmospheric conditions [147]. While there is considerable uncertainty about how climate change will impact the Weddell seal population in the WRS, evidence suggests that the region is cooling and sea ice is expanding, in contrast with other places in Antarctica [76]. However, since the spring of 2016, these strong regional trends have weakened due to the occurrence of anomalously low sea ice and warmer conditions, begging the question as to whether Antarctica is finally feeling the full impact of climate change [151,152]. As a result, Weddell seals in the WRS may have been impacted less in previous decades but are now more recently experiencing extreme change. Nonetheless, the WRS Weddell seal population has been increasing in recent years since its earlier exploitation [40,153]. The continuation of this trend could lead to increased inter- and intraspecific competition and thus result in altered foraging patterns that are still being realized. Our results highlight the WRS Weddell seals as an effective ‘indicator’ of ecosystem change, as merited by CCAMLR, the agency overseeing Ross Sea management [88,89].

## Data Availability

All metadata on the individual animals, their body morphometrics, tracking and diving data have been archived and available at the U.S. Antarctic Program Data Center at the following URL: https://www.usap-dc.org/view/project/p0000661. In addition tracking data were published as part of a larger Antarctic tracking data set and are reported at https://doi.org/10.1038/s41597-020-0406-x [154]. The data are provided in the electronic supplementary material [155].
